# LMTK2 as Potential Biomarker for Stratification between Clinically Insignificant and Clinically Significant Prostate Cancer

**DOI:** 10.1155/2021/8820366

**Published:** 2021-01-05

**Authors:** Alvydas Vezelis, Julija Simiene, Daiva Dabkeviciene, Marius Kincius, Albertas Ulys, Kestutis Suziedelis, Sonata Jarmalaite, Feliksas Jankevicius

**Affiliations:** ^1^National Cancer Institute, Santariskiu Street 1, Vilnius, LT 08660, Lithuania; ^2^Life Sciences Center, Vilnius University, Sauletekio Ave., 7, Vilnius, LT 08412, Lithuania; ^3^Vilnius University Hospital Santaros Klinikos, Santariskių Street 2, Vilnius, LT 08661, Lithuania

## Abstract

A set of prostate tumors tend to grow slowly and do not require active treatment. Therefore, stratification between patients with clinically significant and clinically insignificant prostate cancer (PC) remains a vital issue to avoid overtreatment. Fast development of genetic technologies accelerated development of next-generation molecular tools for reliable PC diagnosis. The aim of this study is to evaluate the diagnostic value of molecular biomarkers (CRISP3, LMTK2, and MSMB) for separation of PC cases from benign prostatic changes and more specifically for identification of clinically significant PC from all pool of PC cases in patients with rising PSA levels. Patients (*n* = 200) who had rising PSA (PSA II) after negative transrectal systematic prostate biopsy due to elevated PSA (PSA I) were eligible to the study. In addition to PSA concentration, PSA density was calculated for each patient. Gene expression level was measured in peripheral blood samples of cases applying RT-PCR, while MSMB (−57 C/T) polymorphism was identified by pyrosequencing. LMTK2 and MSMB significantly differentiated control group from both BPD and PC groups. MSMB expression tended to increase from the major alleles of the CC genotype to the minor alleles of the TT genotype. PSA density was the only clinical characteristic that significantly differentiated clinically significant PC from clinically insignificant PC. Therefore, LMTK2 expression and PSA density were significantly distinguished between clinically significant PC and clinically insignificant PC. PSA density rather than PSA can differentiate PC from the benign prostate disease and, in combination with LMTK2, assist in stratification between clinically insignificant and clinically significant PC.

## 1. Introduction

Prostate cancer (PC) is the second most common oncological disease among men in the world [1]. It still remains as the major malignant disease and the second major cause of death from the cancer between men in developed countries [2]. According to the World Health Organization, around 420000 new PC cases are diagnosed each year in Europe. PC accounts about 7.1% of the total oncological diseases [3].

The prostate-specific antigen (PSA) is a prostate protein discovered and proposed for PC diagnostics in 1970. Nowadays, despite the lack of specificity, it remains the main test for the diagnosis and monitoring of PC [4, 5]. Due to the frequent false-positive results, PSA testing-based diagnosis leads to unnecessary consultations, prostate biopsies, and overtreatment of clinically insignificant cases [6]. Therefore, there is an urgent need for additional PC-specific diagnostic tests empowering separation of clinically significant from clinically insignificant PC [7, 8].

Currently, for prognosis of clinical outcomes, the Gleason score and pathological tumor (pT) stage, along with the PSA test, are used. There are large numbers of studies [9–11] that have suggested a variety of potential prognostic biomarkers in PC. However, these biomarkers are not specifically efficient for the identification of clinically significant PC with a Gleason score ≥7 [12]. Nowadays, the development of genetic analysis technologies allows us to discover new alternative molecular biomarkers that may be used to identify the occurrence of clinically significant PC and predict response to treatment [5].

CRISP3 is an extracellular matrix protein whose expression is regulated by androgens. The protein is involved in prostate carcinogenesis and PC progression and shows predominant expression in PC, but not in benign prostate tissue [6, 13]. However, the exact functional role of this protein in PC remains to be unclear.

MSMB is one of the three major proteins, which in combination with PSA and prostatic acid phosphatase, is secreted by prostate epithelial cells [14]. MSMB protein expression is higher in healthy prostate tissue or benign prostatic hyperplasia (BPH) compared to PC [15]. MSMB protein shows proapoptotic activity and tumor-inhibiting effects through interaction with CRISP3 and other proteins [16]. Moreover, genome-wide association studies have reported the link between a polymorphic variant (rs10993994; *C* > *T*) located in the MSMB promoter region and higher PC risks due to the downregulation of the MSMB [17, 18]. In addition, TT genotype shows associations with the highest susceptibility to develop PC than CC/CT genotypes [19]. Despite the fact that MSMB may be evaluated as a disease genetic risk factor, the role of MSMB in stratification of clinically significant PC cases with rising PSA after negative transrectal systematic prostate biopsy is still unknown [20].

Lemur tyrosine kinase 2 (LMTK2) also known as apoptosis-associated tyrosine kinase (AATYK-2) is a 1503 amino acid protein encoded by the LMTK2 gene [21]. Recent studies revealed interplay between LMTK2 and myosin IV—a regulator of PSA and vascular endothelial growth factor (VEGF) protein expression—as a possible mechanism of antioncogenic action of LMTK2 [22]. The region identified on chromosome 7 may be a new target for drug treatment [20]. A remarkable reduction of LMTK2 protein or transcript levels was reported in PC tissues in comparison to noncancerous tissue or BPH, suggesting involvement of LMTK2 in the development of PC [23, 24]. However, LMTK2 expression in blood samples of PC patients has not been characterized yet.

In the current study, we aimed to investigate the expression level of CRISP3, LMTK2, and MSMB and importance of MSMB rs10993994 polymorphism for the identification of both PC and clinically significant PC in patients with rising PSA levels blood samples.

## 2. Patients and Methods

### 2.1. Patients

The study was performed at the Department of Oncourology of National Cancer Institute (Vilnius, Lithuania) after approval by the Lithuanian Bioethics Committee (no. 158200-16-842–348). Written informed consent was obtained from all patients prior to sample collection. Patient's clinical-pathological data were assessed from medical records at the same institution.

All patients (*n* = 200) who had rising PSA (PSA II) after negative transrectal systematic prostate biopsy due to elevated PSA (PSA I) were eligible to the study. According to the study protocol, blood samples for gene expression analysis were taken, and all patients underwent saturation ultrasound-guided transperineal mapping prostate biopsy (TMPB). Matching asymptomatic controls (*n* = 20) were tested for the same gene expression, and transrectal systemic prostate biopsy was performed. Prostate volume of controls and patients was measured by transrectal ultrasound. PSA density was calculated based on PSA II measurement and prostate volume.

After TMPB cases were divided into three groups: controls (*n* = 20), patients harbouring benign prostatic disease (BPD; *n* = 96), and those diagnosed with PC (*n* = 104). Patients with PC were further subdivided into subgroups of clinically significant PC (csPC; *n* = 50) and clinically insignificant PC (ciPC; *n* = 54). Gleason score ≥7 (3 + 4) with a maximum cancer core length ≥4 mm was considered as csPC [25].

### 2.2. Gene Expression Analysis

Blood samples of PC patients and controls were immediately frozen and stored in −150°C. Total RNA from blood was extracted using “QIAamp RNA Blood Mini Kit” (Qiagen, Germany) according to the manufacturer's instruction. Analysis of CRISP3, LMTK2, and MSMB gene expression was performed by quantitative RT-PCR (qRT-PCR) using KAPA SYBR FAST qPCR Kit (Kapa Biosystems, USA). Gene expression was determined relatively to the expression of housekeeping gene *β*-actin. Each sample was examined in triplicate and calculated following the ΔCt method.

### 2.3. Single Nucleotide Polymorphism Analysis

MSMB SNP rs10993994 was genotyped from the DNA samples using the pyrosequencing system. Pyrosequencing reactions were performed using the PyroMark Gold Q24 reagents (Qiagen, Germany) and the PyroMark Q24 instrument according to the manufacturer's recommendations. The DNA was extracted from patient's blood and amplified, and PCR products were immobilized on streptavidin-coated sepharose beads to obtain single-stranded DNA. After denaturation reactions, the biotinylated single-stranded PCR amplicons were isolated and allowed to hybridize with sequencing primers and finally incubated with DNA polymerase.

### 2.4. Statistical Analysis

The data were analysed using IBM SPSS 21 and STATISTICA 10.0 software. For the analysis of qualitative data, the chi-square test (chi^2^ test) was used. Normal distribution of the quantitative data was tested using Shapiro–Wilk W test. Quantitative data of more than two independent samples were analysed using Kruskal–Wallis ANOVA test, while the data of two independent samples were analysed by Mann–Whitney *U* test. Variables for binary logistic regression were taken according to recommendations of Kim [26]. Binary logistic regression was performed to determine the effect sizes of both genetic and clinical variables for diagnosis of PC as well as csPC. Receiver operating characteristic (ROC) curve analyses were used to compare the performance of genetic and clinical variables in the diagnosis of PC and clinically significant PC by calculating the area under ROC curve (AUC). *P* values less than 0.05 were considered as statistically significant. Materials and Methods should contain sufficient detail so that all procedures can be repeated. It may be divided into headed subsections if several methods are described.

## 3. Results and Discussion

### 3.1. Evaluation of Genetic and Clinical Characteristics as Diagnostic Factors of PC

**Figure 1:**
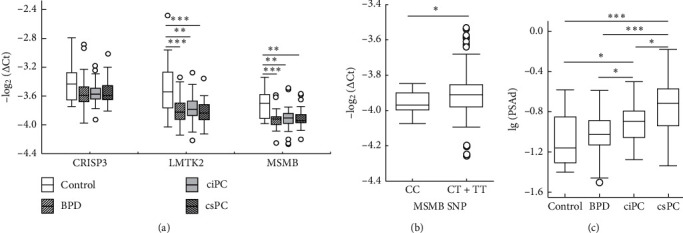
Gene expression (a), MSMB single nucleotide (SNP) (b), and PSA density (PSAd) (c) analysis in control subjects, patients with BPD, and patients with PC stratified to ciPC and csPC. Whiskers of boxplot denote nonoutlier range. Open circles denote the outliers. Expression levels of selected genes were normalised to ACTB (a, b). Arrows above box plots indicate the significant results (*p* value) of post hoc analysis following ANOVA on ranks (a, c) or *p* value of Mann–Whitney *U* test (b).  ^*∗*^*p* < 0.05;  ^*∗∗*^*p* < 0.01;  ^*∗∗∗*^*p* < 0.001; lg(PSAd) denotes logarithm of base 10 for PSA density value, ng/ml.

The main objective of the study was to identify the prebiopsy factors, including genetic and clinical parameters that distinguish BPD cases from PC cases and more specifically -csPC from ciPC with rising PSA levels (Table 1, lines 2–4). For comparison, the expression levels of CRISP3, LMTK2, and MSMB genes were analysed in 20 asymptomatic controls. Genes LMTK2 and MSMB significantly differentiated the control group from both BPD and PC groups (Figure 1(a)), whereas CRISP3 expression differences were insignificant comparing control to both BPD and PC. The expression of all three genes was higher in the control group compared to both BPD and PC. However, LMTK2 and MSMB expression levels were not significantly different between BPD and PC (Table S1).

Moreover, MSMB expression tended to increase from the major alleles of the CC genotype (MSMB CC) to the minor alleles of the TT genotype (MSMB TT) (*p*=0.10, data not shown). Although only seven cases of MSMB TT were identified, the six cases were PC-related (Table S1, line 12). However, the distribution of MSMB CC between BPD and PC was homogeneous (Table S1, line 11). Consequently, the largest MSMB CT group was combined with MSMB TT cases (MSMB CT/TT). The grouping revealed significant differences in gene expression between the MSMB CC and MSMB CT/TT (Figure 1(b)).

Table 1 summarizes clinical characteristics of patients with BPD and PC that were evaluated in the study but not plotted in graphs, and the results show that BPD and PC differ significantly in terms of PSA density. The median of PSA density was significantly higher in PC compared to BPD (Table1, line 5). PSA density (in log10 scale) was significant single diagnostic factor which differentiated PC from BPD with a sensitivity of 65% and specificity of 65% (OR = 46.1 (*p* < 0.001); AUC value was equal to 0.73 (*p* < 0.001)).

### 3.2. Evaluation of Genetic and Clinical Characteristics as Diagnostic Factors of csPC

PSA density was the only clinical characteristic that significantly differentiated csPC from ciPC (Figure 1(c)). The next question was whether the selected genes could reliably distinguish csPC cases from ciPC. No statistically significant differences in CRISP3 and MSMB expression between ciPC and csPC were identified. LMTK2 expression level tended to be higher in ciPC cases compared to csPC (Table S1). Therefore, LMTK2 expression as binary variable (bLMTK2) (Table S1, line 2) and PSA density in lg-scale (lg(PSAd)) were selected for univariate and multivariate binary logistic regression analyses (Table 2). The results of this analysis showed that bLMTK2 and lg(PSAd) as single variables significantly distinguished between csPC and ciPC. In general, combination of both variables remarkably increased the significance of the test. The AUC estimate of the multivariate model was highly significant, and the logistic regression analysis also revealed significant value of the combined test analyses (Table 2).

Although diagnostic procedures and treatment protocols of localized PC are well-established, modern technologies and new knowledge development in cancer biology promote regular changes [27]. Despite the fact that PC is the most commonly diagnosed cancer in males, there are many cases where the disease progresses so slowly that the patient never requires a treatment [28]. The stratification of patients with csPC and ciPC has been at the focus of cancer research for decades and remains a vital issue today [29].

There is no clear consensus on the characteristics of csPC, but Gleason score and tumor volume are considered as the key determinants of disease significance [30]. PC detection through PSA testing still remains controversial and imprecise. The traditional biopsy approach is invasive, costly, and not always answers the purpose [31]. The standard systematic TRUS biopsy remains the general procedure for newly diagnosed PC cases with only a minority (less than 5%) accomplishment by the TMPB [32]. The TMPB method was implemented to improve characterization of cancer type and spatial distribution of cancer cells inside of the prostate tissue, thus increasing PC detection rates. On the contrary, it has been shown that TMPB is associated with increased rates of acute urinary retention, perineal discomfort, and bleeding from urinary bladder due to more extensive sampling of prostate tissue. Moreover, prostate biopsy collection can cause an infection of the urinary tract or prostate that requires treatment with antibiotics and may be associated with difficulty in urinating after the procedure [33]. Thus, despite the fact that TMPB is a much more effective for the PC detection, it is still an invasive procedure with severe complications, compared to blood tasting. Due to fact that invasive testing methods are associated with high cost and delay in diagnosis, the rationality for the development of noninvasive biomarkers is very strong. Due to blood-based biomarkers low risks to the patient and easy access, it represents one of the most attractive methods for the diagnostic evaluation. Personalized molecular screening of noninvasive biomarkers may help identify patients with clinically insignificant prostate cancer in a cost-effective and noninvasive manner [34].

Currently, scientists are focusing on genetic factors and gene expression patterns to find new ways for PC diagnosis and monitoring. In this study, changes in CRISP3, LMTK2, and MSMB expression and MSMB (−57 C/T) polymorphism in blood samples of patients with PC, BPD, and control subjects were detected. We found a statistically significant decrease in LMTK2 and MSMB expression in the blood samples of patients with PC and BPD compared to the control group. PSA density was identified as the only clinical characteristic significantly related to the severity of the disease and showing appropriate diagnostic potential. PSA density significantly differentiated both control subjects and patients with BPD from patients with PC. Thus, initial results have shown that decrease in expression of both LMTK2 and MSMB provides a good distinction between control (with increased PSA levels, but not detected PC disease) subjects and patients with prostate disease. On the contrary, our results indicate that PSA density levels can be used for precise differentiation between patients with BPD and patients with PC. Although the sample size did not allow to reach a high statistical power, our study revealed an increased risk for PC in cases with MSMB TT  genotype that are consistent with results of other studies [14, 35, 36].

For definition of clinically significant disease, we used the recommendations provided by Mark Emberton group at University College London [25]. At Definition I for csPC (Gleason score ≥ 4 + 3 and/or MCCL ≥ 6 mm), only PSA density was significant prebiopsy determinant for cancer risk stratification (data not shown). At Definition II for csPC (Gleason score ≥ 3 + 4 and/or MCCL ≥ 4 mm), both PSA density and LMTK2 were significant prebiopsy determinants for cancer risk stratification, thus indicating that LMTK2 expression is altered at early stages of cancer progression. Despite the fact that LMTK2 expression is not specific for prostate cancer [37–39] due to revealed interplay between LMTK2 and myosin IV—a regulator of PSA—it is widely investigated in prostate cancer [40]. LMTK2 also interacts with other proteins, such as protein phosphatase-1 (PP1C) and inhibitor-2 (Inh2), which are involved in cell division and the cyclin-dependent kinase 5 (cdk5)/*p*35 complex, which plays important role in cell cycle progression. It is therefore likely that that reduction in the amount or activity of LMTK2 may lead to an increase in the proliferative capacity of prostate cells [41, 42]. In this study, patients with ciPC has significantly increased LMTK2 expression level compared to csPC, and this gene can be further investigated as a potential marker for monitoring ciPC progression. Our research results also demonstrate that patients with Gleason score ≥3 + 4 and/or MCCL ≥4 mm has already changed LMTK2 expression in a way favorable for tumor progression, in comparison with ciPC, and the patients should be on active follow-up regimen.

## 4. Conclusions

Taken all together, our data indicate that neither PSA nor PSA variations can separate patients with different diagnoses. Important determinants in diagnosis of PC could be PSA density level and MSMB and LMTK2 expression levels. Furthermore, PSA density level can reliably differentiate PC from BPD. In combination with the LMTK2 measurement, PSA density could be suggested as potential biomarker for identification of PC clinical significance.

## Figures and Tables

**Table 1:** Clinical characteristics of control subjects relative to clinical characteristics of both BPD and PC and clinical characteristics of ciPC relative to clinical characteristics of csPC.

Line no.	Characteristics	Control (*N* = 20)	BPD (*N* = 96)	PC (*N* = 104)	*p* value ^*∗*^	ciPC (*N* = 54)	csPC (*N* = 50)	*p* value ^*∗∗*^
1	Median age, yr, IQR	61, 12.6	64.8, 8.5	65.4, 8.0	0.11	65.4, 8.5	65.6, 8.6	0.71
2	Median PSA I level, first test, ng/ml, IQR	5.3, 4.2	4.7, 1.8	4.9, 2.3	0.74	4.8, 2.1	5.3, 3.8	0.12
3	Median PSA II level, last test, ng/ml, IQR	7.4, 4.7	7.5, 4.7	8.0, 6.3	0.56	7.9, 6.4	9.1, 6.9	0.19
4	Median difference between PSA II and PSA I, IQR	1.66, 3.3	2.58, 3.18	2.54, 3.70	0.52	2.29, 3.08	2.95, 4.06	0.54
5	Median PSA density, ng/ml	0.07, 0.09	0.09, 0.06	0.15, 0.14	<0.001	0.13, 0.1	0.21, 0.15	0.01
6	Gleason score	—	—	80, 24	—	54, 0	26, 24	<0.001 chi^2^
	3 + 3							
	≥3 + 4							

7	Median MCCL, mm, IQR	—	—	3.0, 5.0	—	1.0, 1.6	6.0, 4.0	<0.001

^*∗*^Data were analysed by Kruskal–Wallis ANOVA test for control subjects, BPD, and PC independent samples.  ^*∗∗*^Data were analysed by Mann–Whitney *U* test test for ciPC and csPC independent samples unless otherwise stated. chi^2^, chi-square test; N, group size; IQR, interquartile range.

**Table 2:** Univariate and multivariate logistic regression of the diagnostic factors for csPC cases.

Univariate analysis	Binary logistic regression			AUC	
Variable	OR	*p* value	Sensitivity, specificity	Area (95% CI)	*p* value
bLMTK2:
>−3.80	0.39	0.03	57%	0.61 (0.50–0.73)	0.055
≤−3.80	—	Ref.	67%		

lg(PSAd)	18.5	0.002	60%	0.69 (0.58–0.80)	0.001
			74%		

Multivariate analysis	Binary logistic regression			AUC	
Variables	OR	*p* value	Sensitivity, specificity	Area (95% CI)	*p* value

bLMTK2:
>−3.80	0.28	0.007	64%, 67%	0.74 (0.64–0.84)	<0.001
≤−3.80	—	Ref.			
lg(PSAd)	32.5	0.001			

OR, odds ratio; AUC, area under the receiver operating characteristics curve; CI, confidence interval. Ref., reference group. lg(PSAd) denotes logarithm of base 10 for PSA density value, ng/ml; bLMTK2 denotes the gene expression level as binary variable.

## Data Availability

All data are available on the request (daiva.dabkeviciene@nvi.lt).

## Supplementary Materials

Supplementary Materials Table S1: genetic characteristics for BPD relative to genetic characteristics for PC and genetic characteristics for ciPC relative to genetic characteristics for csPC.
