# Supplementary material for: LMTK2 as Potential Biomarker for Stratification between Clinically Insignificant and Clinically Significant Prostate Cancer
Source: J Oncol. 2021 Jan 5;2021:8820366. doi: 10.1155/2021/8820366 (PMC7803409; doi:10.1155/2021/8820366)
Supplement: Supplementary Materials — Table S1: genetic characteristics for BPD relative to genetic characteristics for PC and genetic characteristics for ciPC relative to genetic characteristics for csPC. [file 8820366.f1.docx]

**SUPPLEMENTARY MATERIAL**

**Table S1.** Genetic characteristics for BPD relative to genetic characteristics for PC, and genetic characteristics for ciPC relative to genetic characteristics for csPC.

| Line No. | Characteristics | BPD  (N = 96) | PC  (N = 104) | P  value | ciPC  (N = 54) | csPC  (N = 50) | P value |
| --- | --- | --- | --- | --- | --- | --- | --- |
| 1 | Median *LMTK2* level,  IQR, N* | -3.82,  0.20, 91 | -3.80,  0.18, 103 | 0.32 | -3.78,  0.18, 54 | -3.82,  0.22, 49 | 0.12 |
| 2 | *bLMTK2* level cut off:  ≤ -3.80, N*  >-3.80, N* | 51  40 | 46  57 | 0.11  Chi^2^ | 23  31 | 30  19 | 0.05  Chi^2^ |
| 3 | Median *CRISP3* level,  IQR, N* | -3.59,  0.19, 94 | -3.58,  0.18, 103 | 0.40 | -3.57,  0.14, 53 | -3.59,  0.20, 50 | 0.80 |
| 4 | *bCRISP3* level cut off:  ≤ -3.58, N*  >-3.58, N* | 50  44 | 49  54 | 0.43  Chi^2^ | 25  28 | 27  23 | 0.49  Chi^2^ |
| 5 | Median *MSMB*  level,  IQR, N* | -3.92,  0.1, 88 | -3.91,  0.1, 103 | 0.28 | -3.91,  0.13, 53 | -3.94,  0.11, 50 | 0.79 |
| 6 | *bMSMB* level cut off:  ≤ -3.92, N*  >-3.92, N* | 45  43 | 51  52 | 0.82 | 25  28 | 27  23 | 0.49  Chi^2^ |
| 7 | Median *MSMB CT* level, IQR, N* | -3.92,  0.1, 76 | -3.90,  0.13, 87 | 0.25 | -3.90,  0.13, 45 | -3.94,  0.12, 42 | 0.74 |
| 8 | Median *MSMB CT/TT* level, IQR, N* | -3.92,  0.1, 77 | -3.90,  0.13, 92 | 0.17 | -3.90,  0.13, 47 | -3.94,  0.13, 45 | 0.93 |
| 9 | Median *MSMB CC/CT* level, IQR, N* | -3.92,  0.1, 85 | -3.91,  0.12, 96 | 0.36 | -3.90,  0.13, 49 | -3.94,  0.12, 47 | 0.86 |
| 10 | *MSMB* SNP:  CC  CT  TT | 10  85  1 | 9  89  6 | 0.18 Chi^2^ | 4  47  3 | 5  42  3 | 0.89  Chi^2^ |
| 11 | *MSMB* SNP:  CC  CT+TT | 10  86 | 9  95 | 0.85 Chi^2^ | 4  50 | 5  45 | 0.90 |
| 12 | *MSMB* SNP:  CC+CT  TT | 95  1 | 98  6 | 0.15 Chi^2^ | 51  3 | 47  3 | 0.75 |

Data was analysed by Mann*–*Whitney U test for two independent samples unless otherwise stated. Chi^2^ is chi-square test. IQR – interquartile range. N – group size, N* - group size of non-extreme range. *bLMTK2, bCRISP3, bMSMB* denote the gene expression level as binary variable.
